# São Paulo School of Advanced Sciences on Vaccines: an overview

**DOI:** 10.1590/1678-9199-JVATITD-2019-0061

**Published:** 2020-04-06

**Authors:** Sara Sorgi, Vivian Bonezi, Mariana R. Dominguez, Alba Marina Gimenez, Irina Dobrescu, Silvia Boscardin, Helder I. Nakaya, Daniel Y. Bargieri, Irene S. Soares, Eduardo L. V. Silveira

**Affiliations:** 1 Department of Clinical and Toxicological Analyses, School of Pharmaceutical Sciences, University of São Paulo (USP), São Paulo, SP, Brazil.; 2 Dipartimento di Biotecnologie Mediche, Universita’ degli Studi di Siena, Siena, Italia.; 3 Department of Parasitology, Institute of Biomedical Sciences, University of São Paulo (USP), São Paulo, SP, Brazil.

**Keywords:** Vaccine history, WHO priorities and challenges, Immune responses, Antigen search, Delivery systems and strategies

## Abstract

Two years ago, we held an exciting event entitled the São Paulo School of Advanced Sciences on Vaccines (SPSASV). Sixty-eight Ph.D. students, postdoctoral fellows and independent researchers from 37 different countries met at the Mendes Plaza Hotel located in the city of Santos, SP - Brazil to discuss the challenges and the new frontiers of vaccinology. The SPSASV provided a critical and comprehensive view of vaccine research from basics to the current state-of-the-art techniques performed worldwide. For 10 days, we discussed all the aspects of vaccine development in 36 lectures, 53 oral presentations and 2 poster sessions. At the end of the course, participants were further encouraged to present a model of a grant proposal related to vaccine development against individual pathogens. Among the targeted pathogens were viruses (Chikungunya, HIV, RSV, and Influenza), bacteria (*Mycobacterium tuberculosis* and *Streptococcus pyogenes*), parasites (*Plasmodium falciparum* or *Plasmodium vivax*), and the worm *Strongyloides stercoralis*. This report highlights some of the knowledge shared at the SPSASV.

## Introduction

The topics discussed at the 2018 São Paulo School of Advanced Sciences on Vaccines (SPSASV) ranged from the most basic knowledge to the highest innovative technologies currently applied in vaccinology. Among them were included: the history of vaccination in mankind, vaccine-derived innate and adaptive immune responses, strategies for the identification of vaccine candidate antigens, vaccination strategies and delivery systems, examples of vaccines against pathogens or cancer, high-throughput technologies to evaluate vaccination outcomes, translational vaccinology and vaccine patents. Below we report the highlights of this event. 

### The history of vaccination and its global impact

In the opening lecture of the SPSASV, Dr. Oscar Bruna-Romero (Federal University of Santa Catarina, Brazil) brought up historical facts about how vaccines were created. Along with sanitation and the discovery of the antibiotics, vaccines have greatly improved the life quality and expectancy of the population worldwide. The first documentation about vaccines is from the 15^th^ century and describes the attempts to prevent smallpox in the Middle East and Asia. Since then, vaccination has evolved from an empirical to a rational and technological approach. Later in the 18^th^ century, Edward Jenner performed the first known vaccine trial. Nearly a century later, Louis Pasteur took advantage of pathogen attenuation in cell cultures to develop the rabies vaccine. This leap of knowledge, combined with the ability to expand viruses in tissue culture, was a crucial step in the prevention of infectious diseases by vaccination. This concept allowed the formulation of vaccines for many infectious diseases, including typhoid fever, plague, and cholera. Subsequently, this progress led to the development of immunostimulatory molecules, denominated adjuvants. Since then, adjuvants have been described as key products for enhancing the potency and efficacy of vaccines and are widely incorporated in their formulations. Furthermore, the ability to produce recombinant proteins significantly heightened the isolation of vaccine candidates from several pathogens during the 1950-60s. Of note, the use of reverse and structural vaccinology combined with synthetic biology has revolutionized the field of vaccination, resulting in the development of novel and safer vaccines. More recently, the application of Systems Biology has also allowed the identification of novel biomarkers that predict vaccine efficacy. Thus, the more the advances made in the vaccinology field, the fewer infection-derived morbidities and mortalities will be observed. Although the World Health Organization (WHO) has estimated that vaccines are responsible for preventing 2.5 million deaths annually [1], a new generation of vaccines is still needed. In addition to infectious diseases, they should also target obesity, Alzheimer’s disease, drug addiction and other emerging public health problems.

### The World Health Organization (WHO) priorities and challenges for the development of novel vaccines

The urgency for novel, safe, widely applicable and more effective vaccines is undeniable, but what are the WHO priorities? Dr. Ricardo Palacios (Butantan Institute, Brazil) introduced the creation of WHO and how it sets priorities in order to finance programs to discover vaccines, drugs and other approaches to fight diseases. Considering that vaccines can rapidly control diseases and do not induce pathogen resistance, the development of novel vaccines, especially for emerging pathogens that affect low-income countries, is arguably a priority for the future. Furthermore, the development of therapeutic vaccines against cancer, autoimmune diseases, Alzheimer’s, chronic infections (HCV, HBV, HPV, HIV), metabolic diseases, allergy, drug addiction, etc., must be in the pipeline as well. Thus, an R&D roadmap is created for each disease, followed by their target product profiles (TPP) including indication, target population, dose regimen, protection duration, administration route, safety and efficacy requirements. Diseases with potential to cause outbreaks, affecting low- and middle-income countries or those with research gaps, are prioritized for the vaccine development. Currently, the priority diseases for WHO vaccine development are: Dengue, HIV, Influenza, Zika, RSV, Malaria, Meningitis, Tuberculosis and Group B Streptococcus [2]. 

Another challenge posed by prioritization is anticipating future needs. Once generated, vaccines, like other pharmaceutical products, have not been easily released to the public, frequently taking 10-15 years from bench to market. Extremely long trials (duration: ≥ 10 years), large budget requirement, regulations and laws that vary from country to country impair straightforward massive vaccination. To overcome these barriers, new models of funding for vaccine development should be stimulated, including the participation of intergovernmental institutions, such as the WHO, governments, industry, academia and philanthropic institutions. Following this idea, the Coalition for Epidemic Preparedness Innovations (CEPI) was conceived in the World Economic Forum in January 2017.

## Vaccine-derived immune responses

Multiple vaccines that have been formulated, ranging from inactivated or live-attenuated formulations to subunits, elicit strong innate and adaptive immune responses. Understanding how these responses are mounted can pave the way for the development of more potent vaccines (and adjuvants) in the future.

### Innate immunity

Considering that adjuvants can stimulate the innate immunity through the activation of inflammasomes [3], Dr. Karina Bortoluci (Federal University of Sao Paulo, Brazil) introduced to the SPSASV audience not only how inflammasomes are assembled and regulated, but also their mechanisms of action. As to infections with the parasite *Trypanosoma cruzi,* the regulation of the inflammasome activation can control the parasite load through autophagy in murine macrophages [4]. For this, PARP1 (a substrate of the NLRC4/caspase-1 axis) exerts an epigenetic control of the iNOS expression [5], critical for the parasite clearance [6]. In a different infection (*Salmonella typhimurium*), inflammasomes drive pyroptosis in response to the cytosolic flagellin with the contribution of cathepsin B [7]. Similar data were also demonstrated for *Legionella pneumophila* [8]. Inflammasome components can be also expressed by cells of the nervous system, and eventually prompt neurological processes [9]. Hence, Dr. Dario Zamboni (University of São Paulo, Brazil) discussed how the innate immune system discriminates between pathogenic and non-pathogenic microbes. Complementing Dr. Bortoluci’s talk, Dr. Zamboni demonstrated that the infection-mediated pyroptosis is also controlled by the expression of Gasdermin-D [10], which induces the pore formation in infected macrophages [11]. Therefore, a better understanding of the mechanisms of action and regulation of these pathways in the innate immune response could help in shaping novel adjuvants.

### Dendritic cells

Some cells of the immune system play a central role in immunity, linking innate and adaptive responses and enhancing immunity levels, such as the dendritic cells (DCs). At the SPSASV, Dr. Silvia Boscardin (University of São Paulo, Brazil) showed how DCs were discovered [12], as well as their functions and relevance to vaccine responses. DCs act as immune sensors in the host environment and, once activated, they upregulate the expression of major histocompatibility complex class II (MHC-II) [13], costimulatory molecules on their surface [14], and secrete cytokines [15,16] to modulate cellular responses [17]. The absence of maturation signals upon antigen contact leads to tolerance [18]. These studies allowed the development of an elegant strategy for new vaccines. In the presence of adjuvants, DC-based vaccines use specific anti-DC antibodies fused to antigens that drive their uptake straight to DCs [19,20]. Curious cutting-edge revelations from DC functions were also highlighted as the first DC cytotoxicity assay and the first anti-DC monoclonal antibody [21], and their participation in the lymphocyte activation [22]. Of note, Dr. Ralph Steinman improved remarkably the knowledge on DCs throughout his career, being awarded the 2011 Nobel Prize for Medicine and Physiology. Thus, the discovery of novel strategies to activate DCs can substantially expedite the development of more potent vaccines against several diseases.

### B-cell development

Antibodies play a pivotal role in providing protection upon exposure to a pathogen. Among the antibody functions, they can neutralize the pathogen entry into the host cell, opsonize antigens and, consequently, stimulate their phagocytosis, activate the complement system or elicit cytotoxicity in the target cell (reviewed by [23]). Currently, most of the vaccines given worldwide induce the generation of antibodies that correlate with protection. Therefore, the characterization of mechanisms that support efficient antibody responses is key to understanding how those vaccines work. Dr. Gabriel Victora (The Rockefeller University, USA) explored how T-cell-independent (TI) and T-cell-dependent (TD) responses impact the kinetics of humoral responses. Moreover, he demonstrated how the memory B cells, as well as the antibody affinity maturation through the process of somatic hypermutation, are influenced by TI and TD responses. The antibody affinity maturation mostly occurs in structures known as germinal centers (GCs) and is able to improve antibody affinity by 100-10,000 fold [24,25]. Whereas the B-cell proliferation concentrates in the GC dark zones, the selection of B cell clones takes place in the GC light zones [26]. In order to explain the details of the GC cellular dynamics involved in the B cell responses, Dr. Victora introduced the “Brainbow System” that allows the visualization of affinity maturation at the single GC level, and updated the model of GC selection [27]. Apparently, only about 5% of the GC expanded B cells in a secondary response are memory cells generated in the first infection. In fact, the majority of the B cells have the phenotype of newly activated naïve cells. Therefore, these data suggest that the diversity of antibody responses increase through repetitive encounters with the same antigen.

## Search for vaccine candidate antigens

Some lecturers at the SPSASV focused on different strategies to identify the best candidate antigens for the development of a vaccine formulation.

### Reverse vaccinology

Until the end of the 19^th^ century, the manipulation of microorganisms to obtain killed or live-attenuated vaccines, currently known as classical vaccinology, was the leading strategy to make a vaccine. Since then, more sophisticated ideas have revolutionized the field, such as the reverse vaccinology [28] or the next generation technologies (synthetic biology, RNA vaccines, structural vaccinology, adjuvants to human immune response). Dr. Rino Rappuoli (Glaxo Smith Kline, Italy) detailed how the concept of reverse vaccinology (a genome-based approach to identify vaccine candidates) has emerged with the development of the Meningococcus B (4CMenB) vaccine. In summary, the entire bacterial genome of *Neisseria meningitidis* was investigated to identify vaccine candidates for meningitis. A total of 570 open reading frames were initially found through *in silico* analysis. Those sequences were amplified and cloned in DNA plasmids for further protein expression. Immunization with 25 of those individual recombinant proteins elicited bactericidal antibodies and were downselected [29]. At the end, 3 potent antigens (two fusion proteins and one single polypeptide - fHbp, NadA and NHBA) were combined with an Outer Membrane Vesicle (OMV) for the final vaccine formulation (reviewed by [30]).

### Antigen discovery

In order to generate more potent vaccines, the strategy for selecting an antigen is critical. Besides the antigen itself, it is important to narrow down what type of response will be induced. Thus, understanding how many and what B- or T-cell epitopes the vaccine must display is critical. Dr. Edecio Cunha-Neto (University of São Paulo, Brazil) focused his presentation on HIV/SIV data. To date, all HIV vaccine formulations that reached a phase III clinical trial did not provide solid protection. Epitope identification for B or T cells remains a major challenge for the development of an HIV vaccine. Ideally, those epitopes should confront viral diversity, cover most of the HLA alleles in the population, induce effector CD4+ T cells, and provide cognate help to CD8+ T cells and antibody responses. Several algorithms employed to predict epitope binding to MHC class I and class II molecules were shown. A direct correlation of the epitope binding prediction and empirical binding to MHC class I and class II molecules was detected through the TEPITOPE algorithm [31]. Subsequently, 18 widely conserved CD4+ T cell epitopes derived from HIV-1 *Gag* were compiled as a polyepitope (HIVBr18) in a DNA plasmid and given to mice. After 3 intramuscular doses of vaccination with this formulation, long-lived Th1 CD4+ and CD8+ T cell responses were induced [32]. HIVBr18 immunization delivered via recombinant adenovirus stimulated even more robust responses than DNA counterpart [33]. In rhesus macaques, the same vaccine formulation elicited broader, stronger and polyfunctional T cell responses in comparison to mice (Edecio Cunha-Neto, personal communication). Therefore, the utilization of available algorithms to define antigen epitopes can significantly optimize the onset of a vaccine. 

## Antigen production and delivery systems

Besides the importance of the antigen selection, the choice of a system for its production and delivery are of utmost significance in vaccine development. Although numerous systems are available for antigen production, most of them change the conformation of the expected protein, impacting directly its protective potential [34]. Also, different outcomes have been demonstrated with the same vaccine administered via distinct routes [35]. Some SPSASV lectures approached how systems of protein production and delivery strategies can impact vaccine-induced immunity.

### The wheat germ cell-free system

Vaccination with recombinant proteins demands a large-scale production of these immunobiologicals, constituting a laborious, expensive, and usually inefficient task. Moreover, the expression of recombinant proteins *in vivo* is highly dependent on their compatibility with the host cells used as the expression platform. Non-mammalian and mammalian systems have been developed to express recombinant proteins. The use of *E. coli* is still the most popular non-mammalian system [36]. In spite of its relative low cost, this well-established system does not allow post-translational modifications, thus dampening the expression of full-length proteins or rendering them insoluble. To overcome these hurdles, Dr. Takafumi Tsuboi (Ehime University, Japan) presented the ‘wheat germ cell-free system’ (WGCFS), which uses the expression machinery of improved wheat germ extracts and is suitable for the expression of proteins derived from bacteria, plants, viruses, parasites, and mammals. It presents flexibility in experimental conditions, requires no codon optimization, allows post-translational modifications and can generate milligram amounts of high-quality proteins. This technology has been successfully applied to find novel malaria vaccine candidates [37-41].

### Heterologous prime-boost strategy

Dr. Arturo Reyes-Sandoval (Jenner Institute, University of Oxford, U.K.) opened up his presentation by highlighting the importance of having a concise structure that integrates Research and Development-conducting units (Wellcome Trust Centre for Human Genetics), Good Manufacture Practice studies for Adenoviruses (Clinical BioManufacturing Facility) and Clinical Trials facilities (Centre for Clinical Vaccinology and Tropical Medicine) for vaccine studies. Taking advantage of this type of integrated structure, the Jenner Institute conducted almost 100 vaccine development projects in the last 10 years, covering aspects from the antigen selection to clinical trials. Vaccines against several tropical disease-causing agents, such as Flavivirus (Zika, Dengue, Yellow Fever, Chikungunya, Mayaro), *Plasmodium* species (Malaria) and *Trypanosoma cruzi* (Chagas disease) have been developed by his group. 

Then, he approached how different formulations can impact the vaccination outcome. Whereas some formulations mainly drive T-cell responses, others represent good options for inducing humoral responses. When different formulations are combined in the same protocol, they are known as heterologous prime-boost regimens, which usually elicit higher magnitudes of both T-cell and antibody responses. In a malaria experimental model of infection, the heterologous prime-boost vaccine regimen induced not only a sustained effector CD8+ T-cell response, but also improved protection in mice [42]. Therefore, heterologous prime-boost vaccine regimens should be more often used.

### Mucosal vaccines

The mucosal immunity portrays the first line of defense against potential environmental injuries [43]. Considering its recurrent contact with food, the mucosal immune system needs to properly discriminate between dangerous and non-dangerous antigens. The induction of tolerance in mucosal tissues is important to avoid mounting immune responses against food, which facilitates the local maintenance of commensal bacteria. Dr. Denise Fonseca (University of São Paulo, Brazil) pointed out that the microbiome composition can exert a significant influence on the regulation of the mucosal immunity through the DC migration from mucosal tissues to lymph nodes [44]. Understanding how immune responses are regulated in these tissues is critical for the development of more potent mucosal vaccine formulations.

Mucosal vaccines present easier administration and better compliance than conventional vaccines, displaying a suitable modus-operandi for mass vaccination. For instance, the administration of the oral poliovirus vaccine reached massive numbers mostly through vaccination campaigns. However, only a few mucosal vaccines are available on the market. In order to be effective, mucosal vaccines must break the tolerance mechanisms, avoid antigen degradation, ensure a potent activation of the immune system and establish a long-lasting immunological memory. In this case, the immunity enhancement has been associated with the expansion of effector T CD4+ cells, especially Th17, and the production of antigen-specific IgA. Different adjuvants or delivery systems for the mucosal vaccination may drive either protective or tolerant immune responses. Dr. Luis Carlos Ferreira (University of São Paulo, Brazil) showed in his talk that mucosal vaccination also induces stronger immune responses at the immunization site and vicinity. Whereas the intranasal immunization efficiently stimulates a protective response in the lungs and the upper respiratory tract, a poor immunogenicity is detected at distant sites, such as the gut. These data warrant research and development of mucosal vaccines against mucosal-infective agents. Of note, it was shown that, similar to other delivery systems, mucosal vaccination is also able to elicit robust humoral responses that can be transferred to infants through breast-feeding [45].

## Targets for vaccines

### Viruses

*Zika*

The Zika Virus (ZIKV) was firstly identified in a sentinel rhesus monkey from Uganda during the 1940s. Since then, few ZIKV infection outbreaks were detected until the explosive ZIKV pandemic that occurred in South America in 2015. Due to the neurological damage and the eventual induction of microcephaly in infected babies, this virus recently occupied the spotlight worldwide. Beyond the onset of microcephaly, ZIKV brain infection can also result in growth impairment, placenta damage, cortical calcifications, eye lesions, and arthrogryposis [46,47]. However, the mechanisms behind this pathology remain incompletely elucidated. Dr. Jean Pierre Peron (University of São Paulo, Brazil) detailed his experimental mouse model of ZIKV-derived microcephaly to study the congenital effects of this viral infection during pregnancy [48]. Radial-glia cells were shown to facilitate brain infection and damage in babies through their contact with the virus via Axl and its ligand, Gas6 [49]. Subsequently, the ZIKV suppresses the interferon response in the central nervous system (CNS), modulating apoptosis and autophagy-related genes [50]*.* Moreover, different mutation patterns of ZIKV particles were related to distinct alterations found in mouse brains depending on the challenged strain (BALB/c and C578/6J).

Multiple attempts to formulate an effective vaccine have been made with ZIKV antigens. Dr. Rafael Larocca (Harvard Medical School, USA) demonstrated that both DNA and recombinant adenovirus-based ZIKV vaccines could afford complete protection against a Brazilian ZIKV isolate in BALB/c mice [51]. Those vaccine formulations conferred protection against challenges with different ZIKV isolates able to replicate in these mice. Experiments with T-cell depletion and passive transfer of purified IgG to mice indicated that vaccine-derived antibodies are key immune correlates of protection. Of note, those vaccines prevented fetus reabsorption and intrauterine growth restriction in a susceptible mouse model (IFN KO). Three different formulations (purified inactivated virus, M-ENV-coding plasmid DNA, and M-ENV-expressing recombinant adenovirus) inhibited the ZIKV replication in rhesus monkeys, but only the recombinant adenovirus induced long-term protection [52]. Furthermore, Dr. Arturo Reyes-Sandoval discussed how the knowledge about the ZIKV replication and dissemination mechanisms can aid in the design of novel vaccines. The elimination of transmembrane domains (TM) from structural viral proteins has improved the secretion of recombinant viruses from infected cells. Those proteins were tested as a vaccine in mice and the induced immune response significantly cleared ZIKV loads [53], supporting the evaluation of this vaccine formulation in future clinical trials.

Besides the preventative vaccination approach, monoclonal antibodies (mAb) have been applied for the treatment of infectious diseases since the 2000s. Regarding this issue, Dr. Esper Kallas (University of Sao Paulo, Brazil) discussed the potency of mAbs cloned from isolated antibody-secreting cells derived from ZIKV-infected patients. More specifically, his group isolated the P1F12 mAb that exclusively neutralizes ZIKV. It recognizes a conformational viral epitope and was used for the development of a ZIKV diagnostic test [54]. Three other mAbs isolated from infected ZIKV patients showed sterile and long-lasting protection against ZIKV in rhesus macaques [55], but could not inhibit vertical transmission [56]. Another available approach is the use of recombinant lentiviruses containing the sequence of a neutralizing mAb as treatment for infectious diseases. Due to the permanent antibody expression, the treatment with a recombinant lentivirus expressing a DENV3-specific nAb protected monkeys against DENV experimental infection (Esper Kallas, personal communication).

*Chikungunya*

Dr. Arturo Reyes-Sandoval talked about the development of a novel Chikungunya virus (CHIKV) vaccine based on the use of a chimpanzee adenoviral vector (ChAdOx). The ChAdOx formulation contains a consensus sequence from Asian and African CHIKV isolates. As a heterologous prime-boost strategy mostly enhances vaccine-derived cellular and humoral immunity, the idea for a CHIKV vaccine consisted of the co-administration of Virus-Like Particles (VLP) and ChAdOx for the priming dose, and VLP and recombinant Modified Vaccine Ankara (MVA) for the booster dose. Whereas high T-cell and antibody responses were observed after the priming in mice, the booster significantly increased those responses as well as the nAb titers against CHIKV. Currently, the ChAdOx vaccine has been tested in Phase I trials with the strategy of dose de-escalation. Preliminary data showed good responses in PBMCs even when administered with ChAdOx at low doses. Another vaccine formulation, based on the Measles Virus (MV) as a vector carrying CHIKV sequences (MV-CHIKV), was effective against CHIKV, but induced lower nAb titers than the ChAdOx formulation. Since the MV vaccination has a wide coverage in Europe, most of MV-CHIKV vaccinees presented T-cell responses against MV, but not for CHIKV. 

*Yellow Fever*

Yellow fever virus (YFV) infections can be severe, with extremely high viral loads systemically disseminated, triggering apoptosis of infected cells. Recent YFV infection outbreaks killed nearly 30% of the symptomatic individuals. Dr. Esper Kallas (University of Sao Paulo, Brazil) presented data obtained using a cohort from the recent YFV infection outbreak that occurred in the perimeter of São Paulo city in 2018. A direct correlation between YFV loads and increased chances of death was found when associated with increased neutrophil numbers, alanine and aspartate transaminases, international normalized ratio, creatinine, and bilirubin levels [57]. To fully recover, the patient requires long-term intensive hospital support, and sometimes even a liver transplant [58]. 

The live attenuated YFV vaccine (17DD) is one of the most efficient vaccines ever developed, providing long-lasting immunity that wanes about 10 years after vaccination. Revaccination can rescue the 17DD-specific long-term immunity [59]. Both innate and adaptive immune responses are elicited by the 17DD vaccination, while nAbs have been defined as the best correlations of protection against this viral infection. 

In spite of its success in preventing YFV infections, the 17DD vaccine does not work as a therapeutic treatment. However, Dr. Kallas also addressed the use of mAbs isolated from 17DD vaccinees for the treatment of YFV infection at the SPSASV. A chimeric mAb (2C9-cIgG) has shown neutralizing activity against YFV, but only *in vitro* [60]. Currently, Dr. Kallas’s group has generated mAbs cloned from isolated antibody-secreting cells of patients derived from the recent Brazilian YFV outbreak as well as from 17DD vaccinees. 

### Bacteria

Disturbingly, a growing number of bacteria are multi-resistant to antibiotic therapy [61]. By 2050, it is estimated that the number of people dying from multi-drug-resistant bacterial infections will be more numerous than those dying from cancer [62]. Dr. Rino Rappuoli explored the current need to develop novel tools to overcome bacterial infections and explained how scientific discoveries, such as antibiotics, hygiene and vaccines, have increased the life expectancy of mankind. For instance, tuberculosis (TB) remains one of the major health problems with almost 25% of all the world population being infected, causing around 1 million clinical cases yearly [63]. The Bacillus Calmette-Guérin (BCG) vaccine was empirically introduced to fight TB in 1921. Despite conferring protection against the severe forms of the acute TB, it fails against chronic infection or pulmonary TB. It is well known that distinct BCG strains differentially influence the immune response against TB [64,65] and have heterogenous prevalence worldwide. It is important in this scenario to identify what bacterial strains are most efficient in eliciting protection against TB and thus chosen to comprise the BCG vaccine. As to immune responses, neither the frequency of BCG-specific T-cells nor the cytokine profile induced by vaccination seem to correlate with vaccine-induced protection against TB in newborns [66]. In this regard, Dr. Nigel Curtis (The Royal Children’s Hospital Melbourne, Australia) revealed that the IFNγ response detected in BCG immunized infants is mostly driven by non-conventional T-cells and NK cells [67].

### Heterologous (non-specific) effects of vaccines

Some vaccines can result in broader benefits beyond direct protection against the diseases for which they were developed [68]. These effects are known as “heterologous”, “non-specific” or “off-target” effects and are different from cross-protective effects, as the latter are elicited against other strains or serotypes of a same pathogen or against a related pathogen. The mechanism behind this phenomenon remains unknown. It has been hypothesized that live vaccines may reduce infant mortality by direct enhancement of innate immune protection, whereas non-live vaccines may do the opposite. There is substantial evidence that these effects are generally stronger in females than in males [69]. Another idea is that the vaccine-mediated heterologous protection would be partly mediated by heterologous effects on the adaptive immunity and by potentiating innate immune responses through epigenetics [70]. Nevertheless, some vaccines present negative heterologous effects, which end up reinforcing anti-vaccine movements. Although this topic remains controversial, much of the evidence is from studies with high risk of bias [71], thus requiring a better understanding of these effects. Dr. Nigel Curtis revealed strong evidence of heterologous effects from the BCG vaccine. It has been documented that BCG vaccination possesses the ability to increase childhood survival in high mortality settings, being stronger in the first 3-6 months after vaccination [72,73]. Several animal studies have associated BCG vaccination with the enhancement of immune responses to unrelated pathogens [68,74]. The Melbourne Infant Study (BCG vaccination to evaluate Allergy and Infection Reduction) demonstrated that the BCG vaccination likely reduced the risks for the onset of some cancers, eczema, food allergies and asthma in more than 1400 babies (http://misbair.org.au/). The BCG vaccination may play a role in the replacement of early life microbial exposure in infants and in reduced incidence of allergies [75]. Moreover, substantial reduction in all-cause mortality was also reported for Measles Vaccination (MV) [76,77]. On the other hand, Dr. Curtis also pointed out that some vaccines are thought to decrease survival in high mortality settings, such as inactivated polio vaccine, diphtheria-tetanus and whole-cell pertussis combined vaccines. The changes in childhood mortality, based on the vaccine schedules, suggest that their heterologous effects may last until a new vaccine is administered. 

### Parasites

The complexity that parasitic infections present when compared to most viral and bacterial infections seems difficult to translate when developing protective vaccines against parasitic diseases. In particular, attempts to reduce the burden of neglected tropical diseases are frequently confined to vector control programs and drug treatments. In the case of parasites that undergo multiple stages of development in the human host, some of them being intracellular stages, complexity frequently resides in triggering an effective immune response against one stage. However, in this case, the induced protection may not cover the response against a different evolutionary stage of the parasite.

### Trypanosomatids

Trypanosomatids are a good example of parasites associated with neglected tropical diseases that affect over 20 million people worldwide. To date, there are no vaccines against those diseases on the market (reviewed by [78]). Their genomes contain multigene families encoding variable surface proteins that hamper the development of an efficient immune response by the host. Dr. Santuza Teixeira (Federal University of Minas Gerais, Brazil) presented a comparative genomic analysis between *Trypanosoma cruzi*, *Trypanosoma brucei* and *Leishmania major*, revealing a conserved gene highly expressed on the parasite surfaces: amastins. Diminishing the expression of alpha-amastin in *Leishmania brasiliensis* led to the generation of attenuated parasites due to an interference in the interactions between amastigotes and the membrane of the parasitophorous vacuole [79]. These results put forward the possibilities for the discovery of novel parasite antigens as virulence factors through modern methodologies, such as CRISPR/Cas genome editing.

In another talk, Dr. Maria Bellio (Federal University of Rio de Janeiro, Brazil) highlighted the importance of Th1 and CD4+ CTL cells in the response to *T. cruzi* infection in a mouse model of Chagas disease. More specifically, the expression of the adaptor molecule MyD88 is critical for the resistance to this infection in mice [80]. It seems to regulate the IL-12 levels as well as the frequency of IFN-gamma-producing CD4+ T cells, essential to control infection [81]. The IL-18 receptor was demonstrated to mediate a critical upstream signal for the MyD88 signaling in T cells for the activation of a protective Th1 response during this infection [82]. In addition, Dr. José Ronnie Vasconcelos (Federal University of São Paulo, Brazil) analyzed the response of CD8+ T-cells during *T. cruzi* infection in mice. These cells mediate the killing of infected cells by granule secretion, apoptosis and cytokine secretion. A heterologous prime-boost vaccination regimen based on DNA and adenovirus platforms carrying the sequence of the Amastigote Surface Protein 2 (ASP-2) protected susceptible mice from a challenge with a pathogenic strain of *T. cruzi*. Besides increasing murine survival, it significantly reduced parasitemia levels and elicited a high frequency of specific CD8+ T cells [83]. Those protective CD8+ T-cells have been characterized as effector T-cells [84,85]. To maintain their efficiency in controlling the parasite loads, these cells need to recirculate but not proliferate. Their migration to the infection site is dependent on the integrin LFA-1 expression [86]. Currently, this vaccination strategy is being studied in preclinical trials in dogs.

### Plasmodium

*Plasmodium* parasites are the causative agents of malaria worldwide. Annually, more than 219 million malaria cases have been estimated with almost half a million deaths [87]. Most of the morbidity and mortality issues are derived from *Plasmodium falciparum* (Pf) infections that are highly prevalent in Africa. On the other hand, *Plasmodium vivax* (Pv) infections, which can also be severe, affect other tropical regions. The mechanisms of Pv pathogenesis remain elusive and there is still no malaria vaccine available. Dr. Fabio Costa (University of Campinas, Brazil) characterized the pathogenicity of Pv infections, highlighting the roles of the antigenic variation, cytoadherence *in vivo* and rosetting [88]. Interestingly, Pv parasites were found in the lung tissues of malaria patients despite being negative for peripheral parasitemia. Moreover, a lower number of schizonts were detected, indicating the possibility of infected red-blood-cell (iRBC) sequestration [89]. The rosetting analysis did not conclusively confirm iRBC sequestration. However, it was demonstrated that plasma factors seem to be involved in rosetting, likely IgM, IgG, IL-10 and IL-6. A transcriptome analysis of leukocytes derived from Pv-infected patients, comparing low and high rosetting patients, highlighted the relevance of phagocytosis-related genes in this process.

To develop an effective vaccine against malaria, it is critical to understand all details about how the immune responses against the parasite are organized. Dr. Ricardo Gazzinelli (Fiocruz, Brazil) reinforced the protective role of antibodies against malaria blood stage parasites, and presented data about the role of cytotoxic CD8+ T cells in this process. Those cells can kill infected cells by detecting parasite-peptides presented by human leukocyte antigen class I (HLA-I). As RBCs, the *Plasmodium* host cells, generally do not express HLA-I, cytotoxic CD8+ T-cells were thought incapable of targeting those cells during malaria infections. However, his group demonstrated that Pv-infected reticulocytes, young RBCs, up-regulate HLA-I expression. Also, CD8+ T cells derived from Pv malaria patients have increased production of toxic granules. The CD8+ T cells colocalized and formed immunological synapses with Pv infected reticulocytes in an HLA-dependent manner [90].

Depending on the desired outcome for a malaria vaccine, different antigens are selected to target distinct stages of the parasite cycle. Antigens derived from the *Plasmodium* pre-erythrocytic and blood stages, or gametocytes, have been selected to inhibit infection, reduce morbidity or block transmission, respectively. Currently, there are a limited number of known and clinically tested malaria vaccine antigens, thus necessitating the discovery of novel antigens as vaccine candidates. Therefore, Dr. Takafumi Tsuboi (Ehime University, Japan) discussed antigen discovery followed by an immunoscreening, based on an unbiased genome-wide analysis of targets for protective antibodies. Using the wheat germ cell-free system described above to express parasite proteins, his group has generated a library of 1827 recombinant malarial proteins. Serum samples of individuals naturally exposed to *P. falciparum* infections from Uganda recognized 938 proteins, of which 128 have been associated with clinical protection [91]. 

### Cancer vaccines

Besides being a strategy directed to prevent infectious diseases, vaccinology has been also applied to inhibit the onset of non-infectious diseases, such as cancer, atherosclerosis, and diabetes. The main challenge of this type of vaccination is to overcome the tolerating condition of the immune system observed during the establishment of these diseases [92]. The establishment of cancer requires the occurrence of multiple processes, such as uncontrolled cell proliferation, induction of angiogenesis, resistance to cell death, evasion of growth suppressors or avoidance of immune destruction. Considering that malignant cells express both self- and non-self-antigens, the interest in developing cancer vaccines able to elicit tumor-specific immune responses has risen. Dr. José Alexandre Barbuto (University of São Paulo, Brazil) exposed the nuances of cancer vaccines by focusing on dendritic cells (DCs). Considering that DCs represent the most efficient antigen-presenting cells and have the ability to tighten up innate and adaptive immune responses, they are crucial players in the development of cancer vaccines [93]. Nevertheless, DCs can have their distribution, numbers, phenotypes and activation status greatly modified by the tumor environment [94]. The adoptive transfer of “healthy” DCs in combination with tumor-antigens (including peptides, proteins, nucleic acids, vesicles or cells obtained from biopsies) might provide an alternative to bypass that problem. Another alternative is the administration of fused DCs-tumor cells that induce tumor-specific immune responses more effectively than a simple mixture of DCs and tumor cells [95,96]. However, the production of those fused cells augments the difficulties of obtaining not only cells from healthy individuals, but also tumors.

Tumor destruction is a process highly dependent on CD8+ T cells as their deficiency leads to an increased tumor incidence in mice [97]. Thus, Dr. Gustavo Amarante-Mendes (University of São Paulo, Brazil) presented new insights into CD8+ T cell-mediated immune responses against tumors. The main mechanism of cytotoxicity used by CD8+ T cells is the degranulation of granzymes and perforin, causing apoptosis in the target cells [98]. A component associated with the killing of tumor cells by CD8+ T cells is Galectin-1, which has been implicated as part of the cytotoxic granule machinery [99]. This molecule has been already described as a regulator of the CD8+ T cell expansion *in vivo* [100], which can eliminate antigen-specific targets *in vivo* [101] by acting through the Fas/FasL axis [102]. Furthermore, the treatment with DNA methyltransferase inhibitors (DNMTi) can enhance the cytotoxic potential of anti-tumoral CD8+ T cells [103], apparently by inducing a mechanism similar to viral mimicry in CD8+ T-cells, facilitating their activation and tumor-suppressing function. 

## Systems vaccinology: The era of high-throughput biology

Dr. Helder Nakaya (University of São Paulo, Brazil) focused his lecture on a current paradigm in life sciences: how can the colossal amount of data generated with new high-throughput technologies be processed and analyzed considering all the evolution from Cellular Biology to Systems Biology? Thus, the ultimate goal of Systems Biology is to obtain data from omics platforms to generate a hypothesis about specific biological conditions and perturbations. More specifically, computational and mathematical models are employed to extract data and validate the system behavior. Corroborating to this plan, the “Top-down systems immunology” utilizes insights from the obtained data to produce hypotheses that can be further tested *in vivo* afterwards. One of the most critical advantages of using Systems Biology is the ability to identify putative predictive gene signatures of immunogenicity, which can be applied to several vaccines. Predictions of the immunogenicity and protective capacity of different vaccines have been successfully explored in recent years [104-106]. Moreover, Dr. Nakaya showed “Systems” approaches combined with novel technologies that his group has developed to identify genes and co-expression gene modules (CEMiTool software [107]). For instance, that tool has been utilized to evaluate the onset of adverse events by vaccines after Ebola vaccination; to track down transmission hotspots for different diseases; among others.

## Translational Vaccinology

Translational vaccinology has bridged the gaps frequently found between vaccine research and advanced clinical studies with the aim of improving public health worldwide. Dr. Andrew Simpson (Orygen Scientific Director) detailed examples of this issue, such as the Flublok vaccine against the influenza virus, clinical trials using the cancer-germline antigen NY-ESO-1, the efficacy of the anti-PD-1 antibody as a cancer therapy and the development of the Poly-ICLC adjuvant. 

The FluBlok consists of the first Flu vaccine based on recombinant protein produced with a baculovirus expression platform system. Recently, it has been licensed by the U.S. Food and Drug Administration (FDA) for vaccination of adults. This vaccine formulation displays higher immunogenicity and lower reactogenicity than standard inactivated influenza vaccines (reviewed by [108]). The NY-ESO-1 is an antigen widely expressed in multiple cancer types that elicit strong anti-tumor humoral and cellular immune responses. Considering its great immunogenicity, there are currently 12 clinical trials based on the NY-ESO-1 antigen as a vaccine candidate and 13 as part of an immunotherapy strategy (reviewed by [109]). In addition, the use of a stabilized form of Poly (I:C), Poly-ICLC (Synthetic dsRNA + polylysine + carboxymethycellulose), also known as Hiltonol or Oncovir, combined with NY-ESO-1 for breast cancer treatment in patients with high risk of recurrence or widespread metastases, had encouraging outcomes [110]. Preclinical trials of vaccine formulations with adjuvant Poly (I:C) or Poly-ICLC against Flu [111,112], Ebola [113], and Malaria [114] have shown augmented cellular responses. The Poly-ICLC treatment alone also demonstrated some degree of protection against some respiratory viral diseases and cancers [115,116], but the mechanisms remain elusive. The anti-PD-1 antibody can rescue the functionality of cancer-derived exhausted T cells. Although side effects have been reported in the context of treatment against PD-L1 highly expressing tumors, improvements have been detected from the combination of this antibody with other treatments (reviewed by [117]).

Another successful example of translational vaccinology was presented by Dr. Ana Paula Fernandes (Federal University of Minas Gerais, Brazil) against leishmaniasis. Visceral leishmaniasis (VL) represents a huge problem in public health as the causative agent infects both dogs and humans. This disease has spread out of control in tropical countries and additional diagnostics and vaccines are strongly needed. Immune responses against the A2 antigen of *Leishmania* can protect nearly 50% of animals in preclinical trials. In this case, the vaccine-derived immunity was associated with the generation of A2-specific antibodies and IFN-gamma secretion. A recombinant A2 protein-based leishmaniasis vaccine (Leish-tec) was constructed and tested in clinical trials. A Phase I / II clinical trial (Safety and Immunogenicity) was successfully performed on dogs with the Leish-tec vaccine with adjuvant of saponin derivatives [118]. A Phase III blinded placebo clinical trial showed 73% efficacy of Leish-tec in preventing VL in an endemic area, allowing the vaccine to be licensed for use in dogs [119]. After 10 years on the market, Leish-tec is currently being tested as a therapeutic vaccine in infected dogs, showing equal efficaciousness and lower toxicity than drugs. Furthermore, trials in immunized non-human primates showed a significant decrease in the parasitic load in lesions induced by leishmaniasis [120]. Additional work on the formulation has been performed to advance Leish-tec vaccination to human clinical trials.

## Design of Clinical Trials

The process of developing a vaccine, followed by scaling-up the antigen production, and fulfilling all regulatory requirements to start a clinical trial can be quite challenging and time-consuming. Dr. Andrew Simpson addressed these issues by detailing how the first worm vaccine, already licensed in Brazil, reached the market. The helminth *Schistosoma mansoni* (Sm) is the causative agent of Schistosomiasis, with nearly 230 million cases worldwide, causing 200 thousand deaths annually (reviewed by [121]). Whereas no vaccine was available, all the background about the immune response against this worm came from animal models. For instance, a primary parasite infection could result in partial immunity against re-infection by Sm in mice. Thus, an attempt to establish a vaccine formulation was performed using adult parasites extracts to immunize mice. That formulation was capable of inducing protection against a challenge comparable to that achieved with live infection [122]. After searching for protective antigens present in the protein extract, a fatty acid binding protein (FABP), termed Sm14, was identified. The vaccination with the Sm14 recombinant protein (rSm14) elicited immunity similar to that induced by the whole extract immunization in mice and rabbits, even without adjuvants [123]. Of note, the analysis of the Sm14 sequence showed high conservation among other disease agents, including veterinary agents, thus facilitating the fundraising for human research. However, existing patents impeded the extended use of rSm14 in humans, which required subsequent investment and research. Considering that the rSm14 vaccine worked in animals, it was adapted for human use. For this, a yeast protein expression system was selected to produce rSm14 [124,125]). In addition, the synthetic adjuvant GLA-SE was selected for incorporation into the final product tested in the clinical trials. Moreover, the creation of a spinoff company helped not only to attract funding and partners, but also to reduce manufacturing costs and quality control testing. Thus, two Phase I clinical trials of the rSm14/GLA-SE vaccination were performed with a Brazilian cohort (total of 20 men and 10 women) without previous exposure to the parasite. Besides safety, Dr. Simpson’s group detected increased cytokines and antibody responses in the vaccinees. The Phase IIa and IIb trials were performed in endemic areas on adults and children in Senegal, Africa, in collaboration with the Pasteur Institute. After the pretreatment with anti-helminth drugs to clear the worms, individuals were vaccinated with 3 doses of rSm14/GLA-SE at 4-week intervals varying the adjuvant dose. Again, safety and immunogenicity were excellent, and the analysis of cellular responses is still ongoing. Apparently, a preexisting infection did not seem to affect IgG titers. A Phase IIb trial, which is about to begin, will enable a first insight into the vaccine efficacy in an endemic area. The development of a commercial vaccine lot required for the next steps has an estimated cost of 10 million dollars. Although a Phase III trial in 5 year-old children is planned, the first published paper on those results would only come out 25 years later.

## Innovation, intellectual property and feasibility

The legal instrument that guarantees the interest of private companies to invest in research and development for new vaccines are the patents. Since patents have allowed a 20-year monopoly on any developed product, all knowledge about them is also critical for the vaccine development. Thus, Dr. Cesar Lopez Camacho (University of Oxford, UK) spoke about how to search for patents, how to patent something, what can be patented, and the legal documentation necessary to complete the process. An interesting consideration was applying for a patent before publishing elsewhere. In order to have a greater chance to progress to clinical trials, novel vaccine candidate studies should be suitable for patents, taking into account the feasibility of a large-scale production. The quality of the patent writing is critical to assure the full protection of the vaccine intellectual property.

## Participant activities

Besides all lectures, the SPSASV 2018 provided space for a poster and a 10 minute-oral presentation by any of the participants. Fifty-three oral-presentations and 2 poster sessions were performed. Presented studies ranged from basic immunology to highly advanced vaccine trials, detailing new strategies or platforms, and targeting multiple pathogens and diseases, such as viruses (Arbovirus, Rotavirus, HIV, Influenza Virus), bacteria (*Mycobacterium, Bordetella, Streptococcus*) and parasites (*Trypanosoma, Leishmania, Plasmodium*), among others. Besides its role in preventing and treating human diseases, vaccination can also be important for animal health and the environment by reducing antibiotic therapy. Two projects presented by attendees aimed to reduce antibiotic therapy in fish farming through vaccination. 

Finally, the participants were further organized into groups and encouraged to present a grant proposal work related to the development of a vaccine against an individual pathogen. Viruses (Chikungunya, HIV, RSV, and Influenza), bacteria (*Mycobacterium tuberculosis* and *Streptococcus pyogenes*), parasites (*Plasmodium falciparum* or *Plasmodium vivax*), and the worm *Strongyloides stercoralis*, were among the selected targets. In order to find feasible modern alternatives for vaccine development, group discussions required several days of collaborative work. Overall, the outcome of each proposal was highly interesting, and we think that all these projects should be brought to fruition.

## Conclusion

To conclude the SPSASV 2018, Dr. Ricardo Gazzinelli emphasized the Brazilian efforts to contribute to vaccine development, especially against neglected infectious diseases through the creation of the National Institute of Science and Technology for Vaccines (NISTV - http://inct.cnpq.br/web/inct-v/pesquisadores/). Besides vaccine development, the goal of this Institution is to improve Brazilian research on basic and applied immunology. The major NISTV targets are Chagas disease, Dengue, Congenital Zika Syndrome, Malaria (transmitted by *P. vivax*) and Visceral Leishmania, which affect millions of people yearly. Moreover, Dr. Gazzinelli highlighted the successes achieved by the NISTV researchers, related to topics such as innate immunity, inflammasomes, adjuvant discovery, cytotoxic CD8+ T cells and host resistance against Chagas disease or Malaria, antigen discovery for *Leishmania* infection, mAb cloning for the identification of novel malaria antigens and others. The NISTV research program is structured into 3 main macro areas (Division of Infectious Diseases and Immunology, Division of Vaccine Technology and Division of Vaccine Development) and sub-areas. This infrastructure allows the cooperation between the different immunology fields, which all convert to Systems Vaccinology studies. Currently, the NISTV partners are Fiocruz, the Federal University of São Paulo, the Federal University of Rio de Janeiro, the University of São Paulo, the Federal University of Santa Catarina, and the University of Massachusetts Medical School.
